# Describing financial toxicity among cancer patients in different income countries: a systematic review and meta-analysis

**DOI:** 10.3389/fpubh.2023.1266533

**Published:** 2024-01-02

**Authors:** Meram Azzani, Wahib Mohammed Atroosh, Deepa Anbazhagan, Vinoth Kumarasamy, Mona Mohamed Ibrahim Abdalla

**Affiliations:** ^1^Department of Public Health Medicine, Faculty of Medicine, Universiti Teknologi MARA, Sungai Buloh, Selangor, Malaysia; ^2^Centre of Occupational Safety, Health and Wellbeing, Universiti Teknologi MARA, Puncak Alam, Selangor, Malaysia; ^3^Department of Parasitology, Faculty of Medicine, Universiti Malaya, Kuala Lumpur, Malaysia; ^4^Department of Microbiology and Parasitology, Faculty of Medicine and Health Sciences, University of Aden, Aden, Yemen; ^5^Department of Microbiology, International Medical School (IMS), Management & Science University (MSU), Shah Alam, Selangor, Malaysia; ^6^Department of Parasitology and Medical Entomology, Faculty of Medicine, Universiti Kebangsaan Malaysia, Jalan Yaacob Latif, Kuala Lumpur, Malaysia; ^7^Physiology Department, Human Biology Division, School of Medicine, International Medical University (IMU), Kuala Lumpur, Malaysia

**Keywords:** direct medical cost, direct non-medical cost, indirect medical cost, catastrophic health expenditure, perceived financial hardship, systematic review, meta-analysis

## Abstract

**Background:**

There is limited evidence of financial toxicity (FT) among cancer patients from countries of various income levels. Hence, this study aimed to determine the prevalence of objective and subjective FT and their measurements in relation to cancer treatment.

**Methods:**

PubMed, Science Direct, Scopus, and CINAHL databases were searched to find studies that examined FT. There was no limit on the design or setting of the study. Random-effects meta-analysis was utilized to obtain the pooled prevalence of objective FT.

**Results:**

Out of 244 identified studies during the initial screening, only 64 studies were included in this review. The catastrophic health expenditure (CHE) method was often used in the included studies to determine the objective FT. The pooled prevalence of CHE was 47% (95% CI: 24.0–70.0) in middle- and high-income countries, and the highest percentage was noted in low-income countries (74.4%). A total of 30 studies focused on subjective FT, of which 9 used the Comprehensive Score for FT (COST) tool and reported median scores ranging between 17.0 and 31.9.

**Conclusion:**

This study shows that cancer patients from various income-group countries experienced a significant financial burden during their treatment. It is imperative to conduct further studies on interventions and policies that can lower FT caused by cancer treatment.

## 1 Introduction

Cancer is the leading cause of death worldwide (1). Cancer cases and deaths continue to increase worldwide in both developed and developing countries. Despite the high cancer incidence rate in developed countries, the mortality rate is higher in developing countries (2, 3). Every year, approximately 400,000 youngsters are diagnosed with cancer (4). The growing number of people diagnosed with cancer places a responsibility on governments to offer services that are suitable, easily accessible, and reasonably priced. However, high-quality services for preventing, detecting, diagnosing, treating, supporting, and caring for those who have survived cancer are challenging to achieve due to multiple influential factors, including unstable politics, inadequately trained cancer care providers, and deficient coordination, in addition to the rising costs associated with cancer treatment (5).

A significant obstacle preventing many cancer patients from receiving therapy and care is the expense of doing so, given the significant geographical variations in patients’ financial capabilities and preparedness to spend money on healthcare and wellness services (6). In the vast majority of low-resourced countries, there is either very little or no universal access insurance coverage for medical care. However, even among insured patients, a significant number are not adequately protected against the expensive requirements of cancer treatment due to the elevated costs of insurance, which include higher co-payments and rising deductibles. Hence, cancer patients typically must pay a significant portion of their treatment costs out of pocket (7). The medical and non-medical expenses of cancer care, which result in a financial burden for cancer patients, are not adequately described in the existing body of research due to the absence of a nomenclature that is consistent throughout the field. Recent research has led to the development of a comprehensive definition of FT, which may be summarized as “The possible consequence of perceived subjective financial distress caused by an objective financial burden” (8). The terms “direct costs” and “indirect care-related costs” refer to “objective financial burden,” but “subjective financial hardship” refers to “material, psychosocial stress, negative feelings, and behavioral reactions to cancer care” (6, 8). FT is a term that is sometimes used interchangeably with terms such as financial or economic difficulties, financial difficulty, financial risk, and economic stress (9). Several studies provided valuable insights into the issue of FT among cancer patients and survivors. Yousuf Zafar (2016) highlighted that FT is a complex problem that affects the quality of life of cancer patients and survivors (10). Tucker-Seeley et al. (2016) build on this, indicating that socioeconomic factors contribute to FT, exacerbating health disparities (11). Arastu et al. (2020) highlighted that financial toxicity can be more prevalent among older adults, and they call for age-appropriate interventions (12). Gordon et al. (2016) found that cancer survivors face additional economic difficulties, such as out-of-pocket expenses and lost of income (13). Baddour et al. (2021) examined the objective and subjective impacts of financial toxicity on head and neck cancer survivors. They emphasize that financial distress not only affects the ability to pay for healthcare but also affects one’s mental health and wellbeing (14).

Ramsey et al. (2013) highlighted that cancer patients are at a greater risk of bankruptcy than individuals without cancer and that the problem of FT can persist beyond cancer diagnosis and treatment (15). Ramsey et al. (2016) further highlighted the connection between FT and early mortality among cancer patients, as financial distress may result in reduced adherence to medical treatments and a lower overall survival rate (16).

In summary, FT is a complex and significant issue for cancer patients and survivors, and its negative consequences can be long-lasting. The studies listed here provide evidence of the widespread occurrence of FT among cancer patients and survivors, indicating the need for policies and interventions to mitigate its effects and improve the quality of life for those affected.

Our study aimed to bridge the current gap in knowledge on FT among cancer patients across different countries with varying income levels. Although recent systematic reviews have examined FT in either low- or high-income countries (17, 18), there is limited comprehensive evidence that explores FT among cancer patients globally.

To address this gap, this systematic review and meta-analysis of existing literature aim to determine the prevalence and measurement of both objective and subjective FT among cancer patients. Our study utilized a rigorous methodology to identify and evaluate relevant studies from diverse sources and synthesize their findings. Through this approach, we aimed to provide a comprehensive understanding of the financial burden that cancer patients face and how it affects their lives.

The current study aims to contribute to the existing knowledge of FT among cancer patients, which will help improve clinical practice and healthcare policies worldwide. Our study will also provide a platform for future research in this area, as we anticipate identifying areas where more research is needed. Ultimately, our findings will assist in addressing the needs of cancer patients and survivors and support the development of effective interventions to mitigate the negative impacts of FT.

## 2 Methods

### 2.1 Search strategy

The systematic review was conducted according to the Preferred Reporting Items for Systematic Reviews and Meta-Analyses (PRISMA) (19) guidelines from 15 August 2022 to 15 January 2023. The protocol was registered in Open Science (Registration DOI: https://doi.org/10.17605/OSF.IO/MUNKG; Supplementary Table S1: PRISMA checklist).

PubMed, Science Direct, Scopus, and CINAHL databases were searched to find articles that reported FT. The search was built based on the research question concerning population/problem (cancer), outcome (financial toxicity), and exposure (healthcare treatment) and (cost of illness), as well as their synonyms (Supplementary File S1: search strategy). There was no limit on the publication year, design, or setting of the study, in order to minimize underreporting bias. In addition, a manual search through the reference list of eligible studies was applied. The search hits for databases are provided in the Supplementary File.

The primary outcome was to find the prevalence of subjective and objective FT among cancer patients. The cost of treatment was also considered a secondary outcome.

### 2.2 Study selection

**Figure 1:**
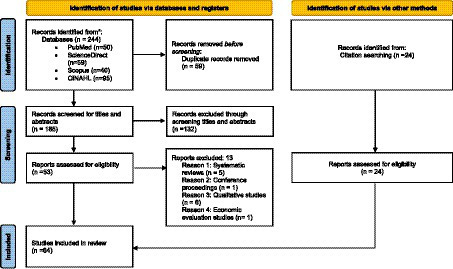
Flowchart of literature review search per Preferred Reporting Items for Systematic Reviews and Meta-Analysis (PRISMA 2020) guidelines.

First, the authors formed a search strategy involving all the relevant keywords based on their knowledge and literature. All search results were transferred to the Endnote X9 software. A total of 244 articles were identified through an online search and 24 articles through a manual search. Then, the duplicate articles were eliminated (20). The titles and abstracts of the remaining 185 articles were screened by two independent reviewers (MMA and VK). Subsequently, a total of 53 articles were retained for full-text review. Disagreements between the two reviewers were resolved by involving a third author (WMA). After a full-text review of the 53 articles, 40 were selected using the on-line search and 24 articles were selected using a manual search. The eligibility of the included articles was agreed upon by all authors. The PRISMA flowchart demonstrated the screening process (Figure 1).

We included studies that are original English quantitative research articles and reported the financial toxicity (objective and subjective) of any type of cancer that were published before 28 August 2022. In addition, studies that reported any cost of cancer, including direct medical, direct non-medical, and indirect costs, were included. This study did not assess the intangible cost as it is difficult to calculate its monetary value. Economic evaluation studies, conference abstracts, reviews, and qualitative studies were excluded. The reasons for these exclusions are as follows: the economic evaluation studies might include the cost of cancer but do not address the primary aim of this review, namely, FT. Conference abstracts do not always present consistent and dependent data. Reviews were excluded because the designed protocols were different from this study; in addition, the outcome evaluation methods were different. Qualitative studies are more of a subjective nature, which cannot be pooled as per the protocol, and their analysis is different from the quantitative data.

### 2.3 Data extraction and quality assessment

The data were presented based on author date, type of cancer, study participants (sample size and sociodemographic characteristics such as age and gender), the prevalence of FT (subjective and objective), cost of illness, tools used to measure the FT, and quality scoring (Supplementary Table S2). The cost of illness is classified into direct and indirect costs. Direct costs are expenses that can be directly and specifically traced to a specific cost object (for example, the medicines consumed by a patient during his/her hospital stay). In contrast, indirect costs are defined as “expenses that cannot be directly linked to a specific cost object (e.g., labour costs) (21). The quality of all included articles was assessed using the *Newcastle* – *Ottawa quality assessment scale* for cohort and cross-sectional studies (adapted for *cross-sectional studies*), which comprises three sections: selection, comfortability, and outcome. The quality score is shown in Supplementary Table S2.

### 2.4 Data synthesis and analysis

Quantitative data were used to find the prevalence of FT. Review Manager 5.3 software was utilized to run the meta-analysis of quantitative data-reported studies. A random-effects meta-analysis was used to calculate pooled data with 95% confidence intervals (CIs). The *I*^2^ index was utilized to assess the heterogeneity among studies, with values classified of ≤25%, 26–50%, and >50% as low, moderate, and high heterogeneity, respectively (22, 23).

## 3 Results

### 3.1 Description of studies

The included studies were carried out worldwide, including in the USA (*n* = 30) (24–53), Europe (*n* = 10) (20, 54–62), Canada (*n* = 4) (63–66), Malaysia (*n* = 4) (67–70), Australia (*n* = 6) (71–76), Brazil (*n* = 2) (77, 78), China (*n* = 2) (79, 80), Iran (*n* = 2) (81, 82), Korea (*n* = 1) (83), Taiwan (*n* = 1) (84), Japan (*n* = 1) (85), and Ethiopia (*n* = 1) (86). A total of 47,964,650 cancer patients participated in a total of 64 studies carried out worldwide, with study samples ranging from 26 to 19.6 million. Out of the 64 studies, 15 studies included participants with any type of cancer (32, 35, 36, 39, 41, 44, 51, 52, 58, 65, 68, 72, 84–86), a mix of 2 or more types of cancers (10 studies) (27, 33, 38, 53, 56, 62, 69, 71, 79, 81), breast cancer (11 studies) (30, 40, 45–47, 50, 55, 66, 76, 82, 83), colorectal cancer (6 studies) (48, 57, 59, 67, 70, 75), colon cancer (*n* = 1) (49), skin cancer (3 studies) (20, 77, 78), lung cancer (2 studies) (54, 80), lung cancer with brain metastasis (1 study) (34), prostate cancer (3 studies) (63, 64, 74), pancreatic cancer (1 study) (28), bladder cancer (2 studies) (29, 31), head and neck cancers (*n* = 3 studies) (43, 60, 61), blood cancer (3 studies) (24, 26, 73), liver cancer (1 study) (42), gynecologic cancer (1 study) (25), and multiple myeloma (*n* = 1) (37).

### 3.2 Measurement of objective financial toxicity

Included research is rarely concentrated, particularly on measurable indicators of FT. Only five studies provided the measurement of objective FT in terms of the prevalence of catastrophic health expenditure (CHE), which was defined as a healthcare cost-to-income ratio of more than 40% in four studies (69, 70, 79, 80) and as the out-of-pocket payment (OOP) that exceeds 10% of total household income in one study (86).

### 3.3 The pooled prevalence of objective financial toxicity

**Figure 2:**
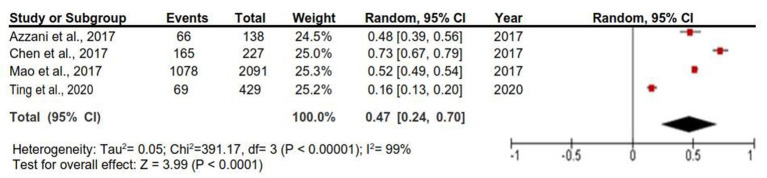
Random-effects meta-analysis of studies that reported the prevalence of catastrophic health expenditure.

A study conducted in Ethiopia reported a 74.4% prevalence of CHE. Two studies were carried out in Malaysia, one among colorectal cancer patients and the other among prostate, bladder, and renal cancer patients, and 47.8 and 16.1% of respondents, respectively, reported having experienced CHE (70, 80). Two studies were carried out in China; one study showed a total of 72.7% of participants experienced catastrophic health spending (69), and the other one showed the prevalence according to the state, where it was 87.3, 66.0, 33.7, and 19.6% in Chongqing, Fuzhou, Beijing, and Shanghai states, respectively (79). We pooled the findings of the last study (79) before including them in the meta-analysis; therefore, the prevalence of CHE in Mao et al. (79) was 51.65%. The last four studies enabled meta-analysis as they used the same method of measuring the CHE. As such, the pooled prevalence of CHE was 0.47 (95% CIs: 0.24–0.70), and the heterogeneity was high (*I*^2^ = 99%) (Figure 2).

### 3.4 Measurement of subjective financial toxicity

In total, 30 studies provided data on subjective FT (24–27, 29, 31, 32, 37, 39, 41, 44–46, 49–52, 58, 59, 61, 62, 64, 67–69, 71, 73, 75, 80, 85). The measures of FT varied widely among the studies. Nine of them used the COmprehensive Score for financial Toxicity (COST) tool, which had 11 items and a score ranged from 0 to 44, where lower COST values indicating higher financial toxicity (25, 26, 31, 37, 46, 68, 71, 73, 85). The median COST score in the included studies ranged between 17 and 31.9. The lowest score was reported among patients with acute myeloid leukemia and the highest among patients with gynecological cancers. Moreover, 10 studies used a 4- to 7-point Likert scale to assess the prevalence of subjective FT (24, 29, 45, 58, 59, 61, 62, 64, 67, 80), where the reported prevalence ranged between 20.9 and 83.7%. In addition, two studies used the median of COST as a cutoff point to assess those with and without FT (25, 26). One study used the Personal Financial Well-Being Scale (PFLBS), which consisted eight items on a Likert scale of 10 points, where 1–4 indicated high FT and LWB, 4.1–6.9 indicated average FT, and 7–10 indicated low FT and high financial WL (69). In addition, one study used four questions with “yes and no” answers to assess the subjective FT; those who responded “yes” to at least one of the four questions were defined as experiencing financial toxicity (44). Ekwueme et al. (2019) described the FT as material hardship and psychological hardship and found it to be 25.3% and 34.3% among the study participants, respectively. Three studies used four questions related to debt incurred, worry about paying bills, and making financial sacrifices as a measure of FT (27, 41, 49). In addition, one study in Australia assessed the FT using three questions related to perceived prosperity, financial strain, and the ability to raise money in an emergency (75) (Table 1).

**Table 1:** Prevalence of subjective financial toxicity in included studies.

**Author, Year**	**Country**	**Cancer type**	**Year of research**	**Sample size**	**Prevalence of FT (%)**	**Tools used**
El-Haouly et al. (2020) (64)	Canada	Prostate cancer	2020	171	22.3%	6-point Likert scale (“not a burden,” “light burden,” “moderate burden,” “considerable burden, but sustainable,” “considerable burden that is difficult to manage and stressful,” and “unsustainable burden”). A categorization was achieved according to the reporting of a moderate/considerable/unsustainable burden (yes/no).
Azzani et al. 2016 (67)	Malaysia	Colorectal cancer	2016	138	20.9%	5-point Likert scale (‘very difficult’, ‘difficult’, ‘somewhat difficult’, ‘not that difficult’, or ‘not difficult at all’). A categorization was achieved according to the reporting of a difficult/very difficult (yes/no).
Chen et al. 2017 (80)	China	Lung cancer	2017	227	83.7%	5-point Likert scale [somewhat, quite a bit, and very much (toxic group) versus not at all or a little bit (non-toxic group) from the COST-PROM questionnaire].
Perry et al. 2019 (45)	USA	Breast cancer	2011–2017	309	37.5% (F strain) 26.1% (FT)	Financial strain is assessed by a four-item checklist that asks participants to indicate whether their income is sufficient to allow them to afford: (1) food and housing, (2) clothing, medicine, and home repairs, (3) going out for a meal and entertainment, and/or (4) a week-long vacation, health permitting. Participants were classified as financially strained if they indicated that they could not afford one or more of the four options. FT was assessed by a 5-point Likert scale: strongly disagree (1) to strongly agree (5), agree and strongly agree are considered as having FT.
Pearce et al. 2018 (58)	Europe	All cancer type	2009–2015	2,931	22%	4-point Likert scale from EORTC QLQ-c30, a little, quite a bit, very much as having FT, not at all as no FT.
Sharp et al. 2018 (59)	Europe	Colorectal cancer	2007–2009	493	41% had financial stress, 39% financial strain, 32% reported both financial stress and financial strain	7-point Likert scale: more difficulty more concern to much less concern, collapse into more difficulty/concern, no change, less difficulty/concern. Financial stress was assessed as the impact of the cancer diagnosis on the household’s ability to make ends meet, and financial strain was assessed as the impact on the individual (i.e., how the respondent had felt about their household’s financial situation since their cancer diagnosis).
Ting et al. 2020 (69)	Malaysia	Prostate cancer, bladder, and renal cancer	2007–2011	429	35.4%	Personal Financial Well-being Scale PFLBS, eight-item Likert scale of 10 points, 1–4 indicate high FT and LWB, 4.1–6.9 indicate average FT, and 7–10 indicate low FT and high financial WL.
Odahowski et al. 2019 (44)	USA	All cancer type	2011	1,419	23.9%	(1) You or anyone in your family had to borrow money or go into debt; (2) You or anyone in your family filed for bankruptcy; (3) You or your family made other financial sacrifices; (4) Unable to cover the cost of medical care visits. those who responded “yes” to at least one of the above questions were defined as experiencing financial hardship.
Ekwueme et al. 2019 (32)	USA	All cancer type	2011–2016	4,753	Material hardship (25.3%) psychological hardship (34.3%)	(1) Material hardship was measured by asking survivors whether they ever had to borrow money, go into debt, file for bankruptcy, or had been unable to cover their share of medical costs. (2) Psychological hardship was considered as being worried about large medical bills.
Bala-Hampton et al. 2017 (26)	USA	Acute myeloid leukemia	2017	26	49.6% Median = 17	COST (COmprehensive Score for financial Toxicity), median 17, less than 17 have distress
Aviki et al. 2021 (25)	USA	gynecologic cancer	2021	89	35% Median = 31.9	COST (COmprehensive Score for financial Toxicity) questionnaire, scored <26 experiencing financial toxicity.
Ehlers et al. 2020 (31)	USA	bladder cancer	2020	226	Median = 28.4	COST (COmprehensive Score for financial Toxicity) questionnaire
Durber et al. 2021 (71)	Australia	Thoracic, breast, carcinoma, skin, CNS, Upper GI, gynecological, head & neck, colorectal & urological cancers	2021	257	Median = 26	COST (COmprehensive Score for financial Toxicity) questionnaire
Rosenzweig et al. 2019 (46)	USA	Breast cancer	March–July 2016	145	Median = 23	COST (COmprehensive Score for financial Toxicity) questionnaire
Yap et al. 2020 (68)	Malaysia	All cancer type	2014–2018	461	Median = 22.0	COST (COmprehensive Score for financial Toxicity) questionnaire
Parker et al. 2022 (73)	Australia	Blood cancer	2020–2021	113	Median = 28	COST (COmprehensive Score for financial Toxicity) questionnaire
Albelda et al. 2019 (24)	USA	Blood cancer	June 2014 and January 2015	171	9% answered not at all for Q1, 6% answered extremely difficult for Q2, and 18% answered not enough for Q3	(1) “How satisfied are you with your family’s present financial situation?” (1 = completely satisfied and 5 = not satisfied at all); (2) “How difficult is it for you/your family to meet monthly payments on your bills” (1 = not difficult at all and 5 = extremely difficult) (3) “How do your family’s finances usually work out at the end of the month?” (1 = some money left over, 2 = just enough money, and 3 = not enough money).
Banegas et al. 2016 (27)	USA	Any type of cancer	4,719	2012	64% reported worrying about having to pay large bills; 34% reported that they or someone in the family had gone into debt because of cancer; 3% of they or their families had filed for bankruptcy; and 40% reported making other financial sacrifices.	(1) Worrying about having to pay large bills related to their cancer, (2) they or someone in the family had gone into debt because of cancer, (3) they or their families had filed for bankruptcy, and (4) making other financial sacrifices
Casilla-Lennon et al. 2018 (29)	USA	Bladder cancer	138		24%	Selecting “agree” or “strongly agree” on the following statement; “You have to pay more for medical care than you can afford” which has 5-point Likert scale options.
Gordon et al. 2017 (b) (75)	Australia	Colorectal cancer	187	January 2010 to Septemper 2011	1 to 0.6% answered as poor in 1st domain at 6 and 12 months, financial strain reported by 15 and 7% at 6 and 12 months, difficult to raise money 41 and 33% at 6 and 12 months	FT questionnaire of three domains: perceived prosperity (prosperous, very comfortable, reasonably comfortable, just getting along, or poor or very poor), financial strain (could not pay utilities on time, could not pay mortgage, or rent on time, sold something, went without a meal, unable to heat home, ask for financial help from friends or family, and asked for financial help from an organization) ability to raise money ($2000) (I could easily raise money, unable/difficult to raise money)
Honda et al. 2019 (85)	Japan	All types (Solid Tumours)	2019	156	Median = 21	COST (COmprehensive Score for financial Toxicity) questionnaire
Huntington et al. 2015 (37)	USA	Multiple Myeloma	Between Aug 18, 2014, and Jan 7, 2015	100	Mean = 23.0	COST (COmprehensive Score for financial Toxicity) questionnaire
Kale et al. 2016 (41)	USA	All types	2011 Medical Expenditure Panel Survey (MEPS)	19.6 million	28.7% reported financial burden.	Cancer Self-Administered Questionnaire (CSAQ) Financial burden was present if one of the following problems was reported: borrowed money/declared bankruptcy, worried about paying large medical bills, unable to cover the cost of medical care visits, or other financial sacrifices.
Rogers et al. 2012 (61)	USA	Head and Neck cancer	January and December 2008	447	54% (at least moderate or large financial burden) 34% (had at least 3/17), and 17% (had at least 5/17).	Self-designed questions about the financial burden and benefits are included in the Cost of Head and Neck Cancer Questionnaire. The severity of burden (no burden, little, moderate, large, or not applicable) in relation to 17 different financial issues, and to say which three had the greatest impact because of their cancer. They were asked the level of difficulty (no difficulty, a little, quite a bit, and very much).
Inguva et al. 2022 (39)	USA	All types	2016–2017		53.7	Cancer Self-Administered Questionnaire of the Medical Expenditure Panel Survey, including FT
Sharp and Timmons, 2016 (62)	Ireland	Breast, prostate, and lung cancer	2008	698	48% reported cancer-related financial hardship, and 32% reported strain	Questions were designed to capture objective and subjective measures of financial difficulties. Seven-level Likert-type scales ranging from much more difficult, very concerned, to much less difficult and much less concerned.
Shankaran et al. 2012 (49)	USA	Stage III colon cancer	2008–2010	284	38% of cancer patients had financial hardships, 23% were in debt, with an average debt of $26,860, and 27% had to sell stocks or use savings or retirement funds	Multidimensional survey instrument was used. FT was assessed through four questions: (1) To pay bills related to cancer treatment, have you had to sell house, borrow money, …etc., (2) Any reduction of income, and how much?, (3) f had to borrow money from other friends or family members, (4) Are you currently in debt due to expenses related to your cancer treatment?
Wheeler et al. 2018 (50)	USA	Breast cancer, all stages	2008 to 2013	2,494 women (49% black, 51% white)	58% of black women, and 39% of white women	A modified model from the National Cancer Institute that describes the direct and indirect contributors to adverse financial impact (decrease of income, financial barrier to care, loss of job, loss of insurance, transportation barrier)
Whitney et al. 2015 (51)	USA	1,209 cancer survivors	2011	All cancer types and stages except skin melanoma	33.2% indicated financial concerns, with 17.9% reporting debt or bankruptcy, 44.0% of working survivors made work adjustments, and 15.3% of which were long-term	A self-administered questionnaire co-developed by the National Cancer Institute (NCI), American Cancer Society (ACS), Centers for Disease Control and Prevention (CDC), National Institutes of Health (NIH), and LIVESTRONG—to provide national estimates of psychosocial, financial, work-related, and other aspects of cancer burden
Yabroff et al. 2016 (52)	USA	1,202	2011	Not specified	28.4% of patients aged 18–64 13.8% of patients ≥ 65 years old.	Material financial hardship was measured by ever (1) borrowing money or going into debt, (2) filing for bankruptcy, (3) being unable to cover one’s share of medical care costs, or (4) making other financial sacrifices because of cancer, its treatment, and lasting effects of treatment. Psychological financial hardship was measured as ever worrying about paying large medical bills

### 3.5 Cost of cancer management

The cost of cancer management was also reported in the majority of included studies (20, 28, 30, 34–36, 38, 42, 43, 47, 48, 54–57, 60, 62–67, 69, 72–74, 76–84, 86).

#### 3.5.1 Direct medical costs

Data on mean direct medical costs from different perspectives were reported in 39 studies in total (20, 28, 30, 33–36, 38, 40, 42, 43, 47, 48, 53–57, 60, 62–67, 69, 72–74, 76–84, 86). The period during which the expenditures were incurred varied widely in the included studies; some studies calculated the cost among cancer survivors (35, 40, 57), in the past 1 month (80), in the last month of life (28), and 2 years after diagnosis (83). The majority reported the annual cost (30, 33, 47, 48, 54–56, 63, 66, 78). Garaszczuk et al. (2022) found that most of the burden is incurred during the first year after diagnosis, and the most costly cancers are lung, colorectal, and prostate (65). The cost perspective varied widely among studies, with the majority using patient perspectives. However, few studies used the provider perspective or the societal perspective. In addition, some considered the cost of health insurance plans. Detailed cost data are shown in Table 2.

**Table 2:** Direct medical, non-medical, and indirect costs in included studies.

**Author, Year**	**Country**	**Type of cancer**	**Year of research**	**No. of patients**	**Direct medical cost**	**Direct non-medical**	**Indirect cost**	**Perspective**
Chu et al. 2008 (84)	Taiwan	Any cancer site (17)	1990–2001	425,294	Highest lifetime cost per case =2,404,000TWD the highest average annual cost per case = 207,000 TWD	NA	NA	National Health Insurance
Andreas et al. 2018 (54)	Europe	Lung cancer	August 2009 and July 2012	306	Mean cost €19,057 (France), €14,185 (Germany), and €8,377 (UK)	NA	Mean costs per patient were €696 (France), €2,476 (Germany), and €1,414	Societal
Dean et al. 2019 (30)	USA	Breast cancer	2015	40	Annual OOP costs = $2,306 compared to $1,090 for those with and without lymphedema	NA	$1,019 compared to $486 for those with and without lymphedema	Patient
Bao et al. 2018 (28)	USA	Pancreatic cancer-stage IV	2006–2011-last month of life	3,825	Median patient OOP ($1,004.8 vs. $228.5) for patients with vs. without chemotherapy	NA	NA	Patient
de Oliveira et al. 2014 (63)	Canada	Prostate cancer	2014	585	Mean OOP costs were $200/year.	NA	NA	Patient
da Veiga et al. 2021 (77)	Brazil	Skin cancer—all stages	NA	NA	Stage 0:359 and 3,135, stage I: 8022 and 39,345, stage 2: 9365–80,036, Stage III: 12,285–556,983, stage IV: 8070–850,686, in public and private, respectively in Reais (R$)	NA	NA	Healthcare provider
Afkar et al. 2020 (82)	Iran	Breast cancer	2020	76	Total mean hospitalization cost (4343.69 USD) Mean (SD) of patient contributions [281.13 (307.22)]	NA	NA	Societal
Azzani et al. 2016 Azzani et al. 2017 (67, 70)	Malaysia	Colorectal cancer-all stages	2016	138	RM 6544.5 (USD 2045.1) for stage I, RM 7790.1 (USD 2434.4) for stage II, RM 8799.1 (USD 2749.7) for stage III and RM 8638.2 (USD 2699.4) for stage IV	RM790(USD246.8)	USD452.2	Patient
Callander et al. 2019 (72)	Australia	All cancer types	2011–2022	25,553	Direct out-of-pocket 380–1,091 among indigenous and non-indigenous people. Indigenous people spent approximately $269 on healthcare co-payments, and $111 in the 7–12 months post-diagnosis. Non-indigenous people spent $359 in the 7–12 months post-diagnosis.	NA	NA	Patient
Chen et al. 2017 (80)	China	Lung cancer	2017 - past month	227	The mean patient costs were $2518.83. The mean total healthcare cost was $2883.44	NA	NA	Societal
Souza et al. 2011 (78)	Brazil	Skin cancer	2007	42,184:non-melanoma skin cancer cases 2,740: skin melanoma cases	The mean annual cost of NMSC/patient was R$1,172 ± 424 in the public healthcare system and R$1,040 ± 664 in the private system. Melanoma: R$13,062 ± 16,848 and R$26,668 ± 42,750, respectively.	NA	NA	HC provider
De Vrieze et al. 2020 (55)	Europe	Breast cancer	2020 per year	194	Total costs per patient were €2248.9. Within these mean direct costs, €1803.35 (80%) was accounted for statutory health insurance and €445.58 (20%) was out-of-pocket expenses for patients.	NA	NA	Health Insurance and patient
Garaszczuk et al. 2022 (65)	Canada	Any cancer (32)	1997 and 2007	2,000,000	CAD$ 26.2 billion in Canada (2021) from a societal perspective; 30% of costs are borne by patients and families. Patients and families’ costs: CAD$ 4.8 billion in 2021.	NA	CAD 2.7 billion	Societal
Hong et al. 2019 (36)	USA	Any cancer	2011 and 2016	655 (2011) 490 (2016)	The mean OOP decreased by $268 (from 384 to 152) after the affordable care act	NA	NA	Patient
Iloabuchi et al. 2021 (38)	USA	Breast, prostate, colorectal cancers, non-Hodgkin’s lymphoma	2016	26,822	Mean cost USD 7764	NA	NA	Patient
Lang et al. 2009 (42)	USA	Liver cancer	1999	392	Annual cost: USD$ 454.9 million, Per patient cost: USD$ 32,907	NA	NA	Healthcare provider
Lauzier et al. 2013 (66)	Canada	Breast cancer	2003	800	Median OOP one year after diagnosis is USD$ 1,002	NA	NA	Patient
Mao et al. 2017 (79)	China	Bronchioles and lung, breast, stomach, colon, and rectal cancers	2008	2091	High total expenditure ($1,228) but lowest OOP payment ($170) among the four cities in China (patients with social insurance)	NA	NA	Health insurance and patient
Murphy et al. 2021 (56)	Europe	Breast cancer, genitourinary, GIT, gynecological	Septrember 2018 to March 2019	238	Annual cost: €53,901, per patient: €226.49 Monthly cost: €7,700, per patient: €32.36	NA	NA	Not covered by HC- fundraising, charitable donations, and volunteer and patient
O Céilleachair et al. 2017 (57)	Europe	Colorectal cancer	October 2007–September 2009	497	Average OOP: €1,589 among colorectal cancer survivors	€510	NA	Patient
Parker et al. 2022 (73)	Australia	Blood cancer	April 2020 to February 2021	113	$14,840 among the whole cohort (medication, allied health, and doctor visit)	$6,700 of the whole cohort	NA	Societal
Sargazi et al. 2022 (81)	Iran	Cervical cancer, Ovarian cancer Endometrial Cancer	2014	10,000	$32 million	NA	$19 million	National HC
Sasser et al. 2005 (47)	USA	Breast cancer	1998–2000	555	Average annual direct costs BrCa ($13,925)	$8,236	NA	Employer Medical Claims
Seifeldin, 1999 (48)	USA	Colon cancer	1991–1994	Mean number of admissions: 237,754 per year	Total hospital charge is $4.5 billion per year (4 years period)	NA	NA	National HC
Ting et al. 2020 (69)	Malaysia	Prostate cancer, bladder, and renal cancer	2007–2011	429	USD$ 9181.1	USD24.8	NA	Government subsidy, medical insurance
Vallejo-Torres 2014 (20)	Europe	Skin cancer	2008 and 2020 (estimate)	8,658 Malignant Melanoma and 73,593 NMSC-Year	Range of £106–£112 million in 2008 and estimated to be £180 million in 2020	NA	NA	NHS
Van Agthoven 2001 (60)	Europe	Head and neck cancers	1994–1996	854	£31,829 per patient	NA	NA	HC provider
You et al. 2019 (83)	Korea	Breast cancer	2003–2011	1,087	Mean cost USD$ 12,108 in 2003–2008) after 2 years of mastectomy	NA	NA	National Health Insurance and patient
Kasahun et al. 2020 (86)	Ethiopia	Any type	2018	352	Mean medical cost: $1978 (median: $1394)	Mean cost: $388 (median: $222)	NA	Patient
El-Haouly et al. 2020 (64)	Canada	Prostate cancer	2020	171	The mean total cost incurred in the last 3 months was $517	USD379.38	NA	Patient
Finkelstein et al. 2009 (33)	USA	Any type	2000–2005	1940	OOP during active cancer stage is USD$1,730 and 1,180 in the follow-up stage.	NA	22.3 days	National Health Insurance and patient
Gordon et al. 2017 (74)	Australia	Prostate cancer	April and June 2013	289	OOP median is AUD$ 8,000	NA	NA	Patient
Gordon et al. 2007 (76)	Australia	Breast cancer	2004–2006	287	Mean cost is USD$ 1,937	NA	Mean cost US$6093	Patient
Guerin et al. 2016 (34)	USA	Brain metastasis among lung cancer patients	January 1, 1999 to March 31, 2013	132	Mean cost USD$86,027	NA	Mean cost USD$8,528	Insurance
Guy et al. 2013 (35)	USA	Any cancer site	2008–2010	4,960	Economic burden of cancer is $16,213 per survivor aged 18 to 64 years and $16,441 per survivor aged ≥65 years.	NA	NA	Patient (OOP), Private insurance, Medicare, Medicaid, and other sources
Jagsi et al. 2014 (40)	USA	Breast cancer	2005 to 2007	1,502	Median out-of-pocket expenses were ≤$2,000; 17% of respondents reported spending > USD$5,000	NA	NA	Patient, Health insurance
Sharp and Timmons, 2016 (62)	Ireland	Breast, prostate, and lung cancer	2008	698	Mean direct medical out-of-pocket costs is EURO€1,491	cancer-related costs (mean = €1,180)	NA	Patient
Zheng et al. 2015 (53)	USA	Colorectal, breast, and prostate cancers	2008 to 2012	Colorectal (non-older adult: *n* = 169; older adult: *n* = 371), breast (non-older adult: *n* = 777; older adult: *n* = 791), and prostate (non-older adult: *n* = 281; older adult: *n* = 889) cancer survivors and individuals without a cancer history (non-older adult: *n* = 95,640; older adult: *n* = 13,792)	Annual excess medical expenditures (for the non-older adult population, colorectal: USD$8,647, breast: USD$5,119, and prostate: USD$358; for the older adult population, colorectal: USD$4,913; breast: USD$2,288, and prostate: USD$3,524).	NA	NA	Patient
Massa et al. 2019 (43)	USA	Head and neck compared to other types of cancer	1998–2015	16,771	Median annual medical expenses (USD$8,384 vs. USD$5,978; difference, USD$2,406; 95% CI, USD$795–USD$4,017)	NA	NA	Patient

#### 3.5.2 Direct non-medical costs

The direct non-medical cost was included in only seven studies (47, 57, 64, 67, 69, 73, 86). Two studies were conducted on colorectal cancer patients (44, 67): one in prostate cancer patients (64), one among patients of three types of cancer, namely bladder, prostate cancer, and renal cancer (69), one among blood cancer patients (73), and one among patients of any cancer type (86). The cost in the last 3 months for prostate cancer was USD$ 379.38 in Canada (64). For colorectal cancer, one study was done in Malaysia and found the cost to be USD$ 246.8 in the first year after diagnosis, and the other one was conducted in Europe and found the cost to be €510 in the three studied years (2007–2009) (57, 67). Ting et al. (2020) found that the cost per patient is USD$ 24.8 in Malaysia, and Parker et al. found that the cost in Australia is AUD$ 6,700 among blood cancer patients (73). In addition, the cost among cancer patients of any type in Ethiopia was USD$ 1,978 (86), and Sasser et al. (2005) found that the annual cost among breast cancer patients was USD$ 8,236. (Table 2).

#### 3.5.3 Indirect cost

A total of eight studies reported the indirect cost of cancer management (30, 33, 34, 54, 65, 67, 76, 81). Total annual indirect costs in Europe per lung cancer patient were as follows: €696, €2,476, and €1,414 in France, Germany, and the UK, respectively (54). It was USD$ 1,019 compared to USD$ 48 for those with breast cancer with lymphadenoma compared to those without lymphadenoma, respectively, in the USA (30). In addition, the cost was USD$ 452.2 among colorectal cancer patients in Malaysia (67). Moreover, 10 years of indirect costs amounted to CAD$ 2.7 billion among patients of any cancer type in Canada (1097–2007) (65). It accounted for USD$ 19 million in Iran in 2014 among gynecology cancer patients (81). In addition, one study reported the work days missed due to disease rather than the cost of productivity lost (33) (Table 2).

## 4 Discussion

This systematic review and meta-analysis describe the prevalence of subjective and objective FT among cancer patients. The included studies are from different income countries and were published between 1999 and 2022. The majority of studies reported only direct medical costs (*n* = 39). Few studies (*n* = 7) reported direct non-medical costs, which include the cost of transportation, food, and accommodation due to disease, and similar findings were observed earlier (17). Moreover, only eight studies reported the indirect cost among cancer patients, which is defined as the cost of productivity loss of patients and their caregivers as a result of cancer. This was also hardly measured previously (17). Even though cancer care expenses are intuitive indications of the financial effects of cancer care, it was challenging to compare the included research cost findings due to the disparate illness course, type, stage, perspective, and the period during which the expenditures were incurred.

The medical expenditure–income ratio may be more suitable than a particular value for medical costs when evaluating cancer-related FT among patients. However, the definition and methods of measurement were contradictory in the included articles. For example, four studies used CHE to measure the household financial burden of healthcare payments, which is a well-established objective tool (87, 88). It is considered that a patient experiences a catastrophic situation when a household’s OOP healthcare expenditure exceeds 40% of the household’s capacity to pay (i.e., effective income remaining after basic subsistence needs have been fulfilled) (89). However, one study estimated the CHE using the Wagstaff and Van Doorslaer approach (90); when households with prior-year cancer patients’ OOP expenses for care exceeded 10% of their total annual household income, it was deemed catastrophic. As such, we only pooled the prevalence of CHE in four studies and found it equal to (47%) (69, 70, 79, 80), which is less than that found in a study conducted in a low-income country (74.4%) (86). However, this value represents only 11% of the included studies, which reflects that the included studies barely focused on measuring the objective FT.

Regarding the prevalence of subjective financial toxicity, it was found to range between 20.9 and 83.7%. There is a huge variation in measuring the subjective FT among the included studies. This finding confirms the earlier observation that there is a lack of accepted definitions of subjective FT (91). Similar findings have been reported by a previous systematic review, which synthesized methods for measuring FT (8, 17). Recently, a few standardized instruments have been developed and validated in an attempt to quantify the financial toxicity of cancer patients. An example of a COST tool is the de Souza (92) instrument, which was developed in 2014 and validated and used in high- and higher-middle-income countries to measure cancer patients’ experiences of financial toxicity. However, it may not apply to lower-middle or low-income countries. The median COST score among included studies [USA (*n* = 5), Australia (*n* = 2), Japan (*n* = 1), and Malaysia (*n* = 1)] ranged between 17 among acute myeloid leukemia and 31.9 among patients with gynecological cancers, in which a low score indicated high financial toxicity (92). Some studies used the median COST score as a cutoff point to define those experiencing FT (25, 26). However, there was a wide variation in the median of included studies, which might require a validation study on COST to standardize the cutoff point to categorize those experiencing FT.

The strength of this study is that it is the first systematic study and meta-analysis to determine the amount of cancer-related financial toxicity and how it has been measured in various income countries. However, this study has several limitations. First, due to the considerable heterogeneity in the outcome measurement utilized in the included studies, our summary of the findings was narrative rather than quantitative (except for CHE). Second, owing to the considerable heterogeneity in the disease period or course during which the costs were incurred, unknowns and inconsistencies in the amount and type of resources included, inflation, and currency rates, we did not synthesize or compare cancer-related expenditures (including medical, non-medical, and indirect costs) across studies.

## 5 Implication and recommendation

Regular clinical evaluations rarely include FT assessments. According to this review, FT affects cancer patients and their families negatively and is common among cancer patients around the world. As a result, in clinical practice, FT in cancer patients needs to get more attention. The evaluation, acknowledgment, and discussion of financial toxicity are crucial milestones. Nurses can work with doctors to analyze patients’ financial burdens and provide information assistance for cancer patients because they have the closest touch with cancer patients and their careers. Therefore, the government, cancer foundations, and other organizations should adopt initiatives such as education and training programs to expand nurses’ awareness of FT assessment and patient assistance programs.

More high-quality research is required, especially from low-income nations, on the FT of cancer. A tool to quantify FT in cancer patients has to be developed and validated in further research.

## Data availability statement

The original contributions presented in the study are included in the article/Supplementary material, further inquiries can be directed to the corresponding author.

## Author contributions

MA: Conceptualization, Writing – original draft. WA: Writing – review & editing. DA: Writing – original draft. VK: Writing – original draft. MA: Writing – original draft.
