# Supplementary material for: Describing financial toxicity among cancer patients in different income countries: a systematic review and meta-analysis
Source: Front Public Health. 2024 Jan 2;11:1266533. doi: 10.3389/fpubh.2023.1266533 (PMC10789858; doi:10.3389/fpubh.2023.1266533)
Supplement: Supplementary file 1 [file Table_1.DOCX]

Supplementary Material

Supplementary file 1

PRISMA checklist

| **Section/topic** | **#** | **Checklist item** | **Reported on page #** |
| --- | --- | --- | --- |
| **TITLE** | | |  |
| Title | 1 | Identify the report as a systematic review, meta-analysis, or both. | 1 |
| **ABSTRACT** | | |  |
| Structured summary | 2 | Provide a structured summary including, as applicable: background; objectives; data sources; study eligibility criteria, participants, and interventions; study appraisal and synthesis methods; results; limitations; conclusions and implications of key findings; systematic review registration number. | 1 |
| **INTRODUCTION** | | |  |
| Rationale | 3 | Describe the rationale for the review in the context of what is already known. | 2,3 |
| Objectives | 4 | Provide an explicit statement of questions being addressed with reference to participants, interventions, comparisons, outcomes, and study design (PICOS). | 3 |
| **METHODS** | | |  |
| Protocol and registration | 5 | Indicate if a review protocol exists, if and where it can be accessed (e.g., Web address), and, if available, provide registration information including registration number. | 3 |
| Eligibility criteria | 6 | Specify study characteristics (e.g., PICOS, length of follow-up) and report characteristics (e.g., years considered, language, publication status) used as criteria for eligibility, giving rationale. | 3, 4 |
| Information sources | 7 | Describe all information sources (e.g., databases with dates of coverage, contact with study authors to identify additional studies) in the search and date last searched. | 3, 4 |
| Search | 8 | Present full electronic search strategy for at least one database, including any limits used, such that it could be repeated. | Appendix A |
| Study selection | 9 | State the process for selecting studies (i.e., screening, eligibility, included in systematic review, and, if applicable, included in the meta-analysis). | 4,5 |
| Data collection process | 10 | Describe method of data extraction from reports (e.g., piloted forms, independently, in duplicate) and any processes for obtaining and confirming data from investigators. | 5 |
| Data items | 11 | List and define all variables for which data were sought (e.g., PICOS, funding sources) and any assumptions and simplifications made. | 5 |
| Risk of bias in individual studies | 12 | Describe methods used for assessing risk of bias of individual studies (including specification of whether this was done at the study or outcome level), and how this information is to be used in any data synthesis. | 5 |
| Summary measures | 13 | State the principal summary measures (e.g., risk ratio, difference in means). | 5 |
| Synthesis of results | 14 | Describe the methods of handling data and combining results of studies, if done, including measures of consistency (e.g., I^2^) for each meta-analysis. | 5 |

Page 1 of 2

| **Section/topic** | **#** | | **Checklist item** | **Reported on page #** |
| --- | --- | --- | --- | --- |
| Risk of bias across studies | 15 | Specify any assessment of risk of bias that may affect the cumulative evidence (e.g., publication bias, selective reporting within studies). | | NA |
| Additional analyses | 16 | Describe methods of additional analyses (e.g., sensitivity or subgroup analyses, meta-regression), if done, indicating which were pre-specified. | | NA |
| **RESULTS** | | | |  |
| Study selection | 17 | Give numbers of studies screened, assessed for eligibility, and included in the review, with reasons for exclusions at each stage, ideally with a flow diagram. | | 4, 5, 6  Figure 1 |
| Study characteristics | 18 | For each study, present characteristics for which data were extracted (e.g., study size, PICOS, follow-up period) and provide the citations. | | Appendix B |
| Risk of bias within studies | 19 | Present data on risk of bias of each study and, if available, any outcome level assessment (see item 12). | | NA |
| Results of individual studies | 20 | For all outcomes considered (benefits or harms), present, for each study: (a) simple summary data for each intervention group (b) effect estimates and confidence intervals, ideally with a forest plot. | | 5, 6 |
| Synthesis of results | 21 | Present results of each meta-analysis done, including confidence intervals and measures of consistency. | | 6 |
| Risk of bias across studies | 22 | Present results of any assessment of risk of bias across studies (see Item 15). | | NA |
| Additional analysis | 23 | Give results of additional analyses, if done (e.g., sensitivity or subgroup analyses, meta-regression [see Item 16]). | | NA |
| **DISCUSSION** | | | |  |
| Summary of evidence | 24 | Summarize the main findings including the strength of evidence for each main outcome; consider their relevance to key groups (e.g., healthcare providers, users, and policy makers). | | 6-16 |
| Limitations | 25 | Discuss limitations at study and outcome level (e.g., risk of bias), and at review-level (e.g., incomplete retrieval of identified research, reporting bias). | | 17 |
| Conclusions | 26 | Provide a general interpretation of the results in the context of other evidence, and implications for future research. | | 18 |
| **FUNDING** | | | |  |
| Funding | 27 | Describe sources of funding for the systematic review and other support (e.g., supply of data); role of funders for the systematic review. | | NA |

*From:*  Moher D, Liberati A, Tetzlaff J, Altman DG, The PRISMA Group (2009). Preferred Reporting Items for Systematic Reviews and Meta-Analyses: The PRISMA Statement. PLoS Med 6(6): e1000097. doi:10.1371/journal.pmed1000097

For more information, visit: **www.prisma-statement.org**.

Page 2 of 2

| **Supplementary File 2: Data extraction table** | | | | | | | | |
| --- | --- | --- | --- | --- | --- | --- | --- | --- |
| **NO.** | **Author year** | **Country and setting** | **sampling** | **Cancer Type and stage** | **Tools to measure financial toxicity** | **results** | **conclusion** | **scoring** |
| 1. | Chu et al. 2008 | Taiwan, National  Cancer Registry database, 1990 to 2001 | Sample size: 425,294 | Oral cavity,  nasopharynx, oesophagus, stomach, colorectum,  liver, gallbladder and extrahepatic bile duct, pancreas,  lung, leukaemia, skin, breast, cervix uteri,  ovary, prostate, bladder, kidney and other  urinary organs. | Objectives: to estimate lifetime health expenditure for patients with 17 types of major cancers.  They used Method for extrapolation of life  Expectancy to get lifetime cost incurred by National health insurance | Medical cost:  highest lifetime health expenditure per case (2,404,000TWD)  the highest average annual health expenditure per case (207,000 TWD): leukaemia  highest total lifetime health expenditure  (5046 million TWD): breast cancer followed by Colorectal cancer (4995 million TWD) | The proposed method is a feasible way of estimating lifetime health expenditure for cancer patients even under high censoring rates. This would be helpful for the cost-effectiveness assessment of cancer  prevention programs and policy planning | 4 |
| 2. | Andreas et al. 2018 | France, Germany and the United Kingdom | Sample size: 306  aged ≥18  August 2009 and July 2012  Response rate:58%  Median follow-up: 26 weeks  Survey-based study | Patients with resected non-small cell lung cancer  (NSCLC) | Direct, indirect, and patient out of pocket expenses and National annual costs  Direct cost (for example local hospital  emergency care and general practitioner visits), indirect costs (lost  productivity for patients and caregivers), patient out-of-pocket expenses  (non-reimbursed transportation and childcare) | Annual cost estimates€478.4 million in France, €574.6 million in Germany and €325.8 million (£267.1  million) in the UK  The annual indirect national cost was approximately €35.7 million in France, €111.5 million in Germany,  and €79.4 (£65.1) million in the UK. Annual out-of-pocket expense estimates were reported for Germany (approximately €3.4 million  for non-reimbursed transportation) and the UK (€2.9 million [£2.4]  for non-reimbursed transportation and €1.08 million [£887,649] for  childcare) only. | In all three countries,  the economic burden was substantial. large annual national costs, mainly incurred during disease progression | 5 |
| 3. | Dean et al. 2019 | USA, In 2015, 129 breast cancer survivors from Pennsylvania and New Jersey | 129 patients | Compares out-of-pocket costs for breast cancer survivors with and without lymphedema | The survey-based study, prospective  monthly out-of-pocket cost diaries over 12 months | Annual adjusted health-related out-of-pocket costs excluding productivity losses  totalled $2306 compared to $1090 (p = 0.006) for those without lymphedema, or including productivity losses, $3325 compared  to $2792 | Long-term cancer survivors with lymphedema may face up to 112% higher out-of-pocket costs than those without  lymphedema, which influences lymphedema management, and impacts savings and productivity. | 4 |
| 4. | El-Haoulyet al. 2020 | Quebec , Canada, an outpatient clinic of the Centre Hospitalier de Rouyn-Noranda | Sample size: 171 (RR: 68.4%)  mean  age was 68.73 | Prostate  cancer | Prostate cancer-related direct medical and indirect costs (out-of-pocket costs) from the patient’s perspective (last 3 months) and perceived financial burden assessed using a six-point Likert  scale | The mean total  The cost incurred in the last three months was $517 and 22.3% reported a moderate, considerable, or unsustainable burden | Prostate  cancer patients incur out-of-pocket costs even if they were diagnosed many years ago and the  the perceived burden is significant. | 5 |
| 5. | Ekwueme et al. 2019 | USA | Sample size:  4,753 cancer survivors aged 18–64 years, 2011–2016 Medical Expenditure Panel Survey (MEPS). | Not specified  All cancers | Out-of-pocket spending was estimated in two ways: 1) annual out-of-pocket spending in 2016 dollars and 2) high annual out-of-pocket burden (defined as spending >20% of annual family income on medical care).  Perceived FT is measured in material and psychological domains. Material hardship was measured by asking survivors whether they ever had to borrow money, go into debt or file for bankruptcy or had been unable to cover their share of medical costs. Psychological hardship was considered as being worried about large medical bills. | Average annual out-of-pocket spending per person was significantly higher among cancer survivors ($1,000) than among persons without a cancer history ($622). Financial hardship was common; 25.3% of cancer survivors reported material hardship (e.g., problems paying medical bills), and 34.3% reported psychological hardship (e.g., worry about medical bills). | These findings add to accumulating evidence documenting the financial difficulties of many cancer survivors. Mitigating the negative impact of cancer in the United States will require implementing strategies to alleviate the disproportionate financial hardship experienced by many survivors. | 5 |
| 6. | Bao et al. 2018 | USA | A retrospective, cohort study of 3,825 patients 66 years or older when diagnosed with Stage IV pancreatic cancer in 2006–2011, | Stage IV pancreatic cancer | Costs (both Medicare payment and patient out-of-pocket costs) in the last 30 days of life associated with chemotherapy use | Chemotherapy use is associated with a more than 50% increase in patient out-of-pocket costs for care ($1,311.5 vs. $841.0, p<0.001) in the last 30 days of life. | Financial burdens should be heightened and should be taken into account by clinicians upon deciding on chemotherapy | 4 |
| 7. | de Oliveira et al. 2014 | Ontario, Canada | 585, the mean age was 73 years | Long-term (2–13 years) prostate  cancer survivors | Out-of-pocket costs | Mean OOP costs were $200/year | Out-of-pocket costs are generally manageable for long-term PC survivors  but can be a significant burden mainly for lower income  patients. | 4 |
| 8. | da Veiga et al.2021 | Brazil | Sample size not mentioned | Cutaneous melanoma, cost-of-illness study (public and the private health system) | Cost-of-illness study direct medical cost in a bottom-up analysis, from diagnosis up to 3 years of follow up  Direct medical and costs | Stage 0:359 and 3135, stage I: 8022 and 39,345, stage 2: 9365 -80,036, Stage III: 12,285 -556,983, stage IV: 8070 - 850,686, in public and private respectively  Using Brazilian currency: Reais (R$), R$1,0 = USD0,191,898 | The cost of treatment in private is much more than that in public hospitals reach to 90% in metastatic stage | 3 |
| 9. | Afkar et al. 2020 | Iran | 76 breast cancer | Breast cancer | Prevalence-based cost-of-illness study  Direct cost | Total direct medical cost: 4343.69 USD), the highest cost goes for hoteling | The cost of breast cancer is substantial | 4 |
| 10. | Azzani et al. 2016 | Malaysia, UMMC | Sample size:138 | Colorectal cancer patients | Direct out-of-pocket  non-medical  indirect cost data  The patient’s perceived level of financial difficulty  and types of coping strategies were also explored. | The total 1-year patient cost (both direct and indirect)  increased with the stage of colorectal cancer: RM 6544.5 (USD 2045.1) for stage I, RM 7790.1 (USD 2434.4) for stage II, RM 8799.1 (USD 2749.7) for stage III and RM 8638.2 (USD 2699.4) for stage IV. FT=20.9% | Despite the high subsidisation in public hospitals,  the management of colorectal cancer imposes a substantial  the financial burden on patients and their families. | 5 |
| 11. | Callander et al. 2019 | Australia | n = 25,553 Indigenous and non-Indigenous | All cancer types, no stage mentioned | Direct out-of-pocket patient co-payments and time opportunity costs (length  of hospital stay) incurred by Indigenous and non-Indigenous, one year after diagnosis (2011-2022), using database population | Direct out-of-pocket 380-1091 among indigenous and non-indigenous populations respectively. For both Indigenous and non-Indigenous people with cancer, there was a greater number of hospital admissions (3.9 and 4.6, respectively) | There is a strong need for greater transparency in patient co-payment amounts for healthcare to allow patients to tailor care to ensure they are receiving the best value for their own circumstances. | 4 |
| 12. | Chen et al.2017 | China  Shanghai Chest Hospital, | 227 | Lung cancer (all stages) | Objective: catastrophic health spending (cost-to-income ratio of more than 40%,)  Subjective: perceived financial difficulty  direct medical costs, | The mean of direct medical costs in the past month was  $2518.83.  The mean of total healthcare cost in the past month was $2883.44  Catastrophic health spending:  72.7% of the participants, Financial difficulty was perceived in 83.7% of the participants | Objective and subjective indicators  of financial burden are indicators to identify the patients who may  need additional assistance. Communication on deciding on  cost-effective treatments can be facilitated. | 4 |
| 13. | Souza et al. 2011 | São Paulo, Brazil | 42,184 non-melanoma skin cancer (NMSC)  2,740 cases of skin melanoma | Skin cancer, all stages | Direct medical cost in the public and private sectors in 2007 | The mean annual cost of the treatment of NMSC per patient was R$1,172 ± 424 in the public healthcare system and R$1,040 ± 664 in the private system. These values are very much lower than those found for the treatment of melanoma: R$13,062 ± 16,848 and R$26,668 ± 42,750, respectively. | The high number of cases of non-melanoma skin cancer in Brazil,  represents a financial burden to the public and private healthcare systems of around R$37 million and R$26 million annually, respectively. | 4 |
| 14. | De Vrieze et al. 2020 | Belgium | 194 patients | Breast cancer-related lymphedema  (BCRL) | the direct healthcare costs  related to BCRL and its treatment | Total direct healthcare costs per patient were €2248.93 on average during the entire period of 3 weeks of intensive treatments and 12 months of maintenance decongestive therapy. Within these mean direct costs, €1803.35 (80%) was accounted for statutory health insurance, and €445.58 (20%) was out-of-pocket expenses for patients. | The present study indicates that the price tag of BCRL treatment in Belgium is high not only for health insurance but also for the patients | 4 |
| 15. | Ehlers et al. 2020 | USA | 226 patients, median age was 68 years with 64% male, 83% married, | Bladder cancer | Financial toxicity (FT) is assessed using the COST (COmprehensive Score for financial Toxicity) questionnaire | Mean COST was 28.4(range 0-44) low score means worse FT | Most patients preferred to discuss treatment costs with their  bladder cancer provider | 4 |
| 16. | Bala-Hampton et al. 2017 | USA, National Cancer Institute | 26 | Acute leukemia | A cross-sectional -perceived economic hardship among a cohort of patients at least 6 months postdiagnosis of acute myeloid leukaemia (AML), using the Comprehensive Score for Financial Toxicity (COST-PROM), higher score means less hardship | 26 of 32 eligible patients. Patients with AML reported high levels of economic hardship and distress | It is essential to develop and evaluate interventions that foster patient-clinician communication and referral for services related to both financial need and psychosocial distress. | 4 |
| 17. | Aviki et al. 2021 | USA | 89 gynecologic cancer patients | Gynaecologic cancer patients | Assess financial toxicity [Comprehensive Score for Financial Toxicity (COST)], cost-coping strategies, and preferences for intervention | Median COST score was 31.9 (IQR: 21-38); 35% (N = 30) scored < 26, indicating they were experiencing financial toxicity. | The study provides insight into patients' needs for targeted interventions to mitigate the harm of financial toxicity | 4 |
| 18. | Murphy et al. 2021 | Ireland, East Galway & Midlands  (EG&M) Cancer Support Centre | A total of 238309, 66%  female, 29% male, and 5% unknown. | Breast cancer patients  (47%), genitourinary (15%), gastrointestinal cancer patients  (13%) and finally gynecological (8%) | Objective estimates the economic costs of the resources used to deliver the services to cancer patients at EG&M support centre  Cost analysis tool:  A micro-costing approach, employing standard methods, was used to estimate the costs of running the service, in line with national guidelines  The resources used were identified and measured from the service’s records. | The 1-year cost for the health resources services was €53,901, which  averages to €7700 per month in total, while the average cost of health resources per patient of €226.49  the two full  time volunteers are €6850, bus driver €3243, bus running costs  €1556 (including rent, fuel, and tolls) and lunches €6796 | Evidence on costs and cost-effectiveness is sparse.  Given estimates that by 2020, 1 in 2 will get cancer in their  lifetime, the demand for community services  complementing and supporting hospital care is expected to  increase. | 6 |
| 19. | O Céilleachair et al. 2017 | Ireland, National Cancer Registry | All cases of primary, invasive colorectal cancer in Ireland diagnosed October 2007–September  2009,  questionnaires were returned (response rate = 39%).  N=497 | Colorectal cancer, stage I, II, II, IV, and unknown. | Investigate out-of-pocket costs (OOPCs) incurred by colorectal (CRC) medications and indirect  costs). | The average OOPC was €1589. | Greater attention  should be paid for the development of services to help  survivors manage the financial and economic burden of  cancer. | 6 |
| 20. | Parker et al. 2022 | Australia,  Two metropolitan tertiary health services based in Melbourne, Australia. | A total of 113 participants, 53 females and 60 males.  Most participants were aged between 40 and 65 years (48 %). | Lymphoma (11%), leukaemia (62 %),  and Multiple Myeloma (27%)  Grades: Grade 0 to Grade 3. | Objective: to measure financial toxicity experienced by patients using patient-reported outcome Comprehensive Score for financial toxicity (COST)  Financial toxicity outcome measure | In this cohort, the mean COST score was 27 (±SD11.83) and a median score of 28 (range 0− 44). | Financial toxicity is present in Australian haematology patients and those at higher risk may be patients of working age, those without private health insurance | 6 |
| 21. | Odahowski et al. 2019 | USA  Utilized data from the 2011 Medical Expenditure Panel Survey (MEPS) | Sample size: 1419 cancer survivors with 275; 110 males and 165 females residing in a rural MSA  Age ranges from 18- 85 years | All types of cancer including non-melanoma skin cancer survivors | Objective: the study aimed to examine financial hardship  A measure of financial hardship using a survey consisted of four questions and one question regarding financial worry. | More rural cancer survivors reported financial hardship than urban survivors (23.9% versus 17.1%). | Improving patient-provider communication through decision aids and/or patient navigators may be helpful to reduce financial hardship and worry regardless of rural-urban status. | 7 |
| 22. | Perry et al. 2019 | USA,  Participants with a history of breast cancer were recruited using the NIH ResearchMatch database. | Three samples of women with breast cancer (Total N=309; Sample 1: N=55; Sample 2: N=120; Sample 3: N=134) participated in this research, conducted from 2011–17.  The inclusion criteria were having a current or past diagnosis of breast cancer, being female, and being at least 18 years of age. Patients unable to read English were excluded. | Breast cancer | Objectives: To examine the association between financial strain and key elements of the physical and emotional quality of life among women with breast cancer.  1.Financial strain using a 4-item checklist  2.Financial toxicity was assessed, in Sample 3 only, using an item from the financial domain of the Patient Satisfaction Questionnaire rated from 1 (strongly disagree) to 5 (strongly agree): “I have to pay for more of my medical care than I can afford”. The presence of financial toxicity was determined if participants rated the item a 4 or 5 (agree or strongly agree). | 37.5% of the total sample reported financial strain.  Financial toxicity was present in 26.1% of Sample | The financially strained women with breast cancer experienced the worse emotional and physical quality of life. | 5 |
| 23. | Rosenzweig et al. 2019 | Pittsburgh, Pennsylvania  The University of Pittsburgh & UPMC Magee-Women’s Hospital | 145 women with metastatic breast cancer received care at an urban outpatient breast cancer clinic and the Women’s Cancer Program at UPMC Magee-Womens Hospital from March to July 2016. | Metastatic breast cancer | Objectives: To determine the incidence of financial toxicity among women with metastatic breast cancer,  Design: A cross-sectional  Financial toxicity was evaluated using the 11-item COST tool | Study participants had a mean age of 58.1 years  the mean COST value was 22.6 (SD = 11.5, median = 23, range = 0–44) | Financial toxicity is common among women with metastatic breast cancer and more common among low-income women with the disease. | 7 |
| 24. | Pearce et al, 2018 | Netherlands,  Data was pulled from the  The Patient Reported Outcomes Following Initial Treatment and Long-term Evaluation of Survivorship (PROFILES) registry,  age (between 18 and 65 years). | A total of 2931 with an even number of men and women.    Mean age of 55 years (range 18 to 65). | Diverse cancer types:  One-third of the sample (33%) had haematological cancer, and 31% had colorectal cancer. The remaining participants had gynaecological cancer (12%), basal cell carcinoma (9%), thyroid cancer (7%), prostate cancer (7%) and melanoma (2%). disseminated (2009 and 2015). | Objective: prevalence of, and factors associated with, financial toxicity among cancer survivors  Method: FT measured by: ‘Has your physical condition or medical treatment caused you financial difficulties in the past week?’ Responses of ‘A little’, ‘Quite a bit’ or ‘Very much’ were combined as having experienced financial toxicity, and responses of ‘Not at all’ were considered to not have financial toxicity. | Out of 2931, 22% reported financial toxicity | Unemployment is significantly associated with financial toxicity and those with limited financial resources are most at risk. | 6 |
| 25. | Durber et al. 2021 | Australia  Department of Medical Oncology at Sir Charles Gairdner Hospital, Perth, Western Australia | N=257  Female=119  Male=138  ≤50=58  51-64=77  ≥65=122  No details on ethnic groups | Thoracic, breast, carcinoma, skin, CNS, Upper GI, gynaecological, head & neck, colorectal & urological cancers were included  Stages (I-IV, upstaged) | Cross-sectional study in a single centre  questionnaire pack- FT-measured via the FACT COST tool.  The test-retest study was also conducted. Primary endpoint -degree of financial toxicity experienced via the COST questionnaire. | Financial toxicity  was greatest in younger participants, those with more inpatient admissions, those with a change in employment status following diagnosis, and those in the lowest income  quintile. Financial toxicity was associated with worse health-related quality of life, and greater depression and anxiety. | Increased financial toxicity is associated  with worse psychological well-being and certain patient demographics. | 5 |
| 26. | Garaszczuk et al. 2022 | Canada  The Canadian Partnership Against Cancer, Toronto, ON | Patients diagnosed with primary cancer between 1997 and 2007  OncoSim-All Cancers model projected around 2,000,000 people | 31 cancers: oral cavity,  oropharynx, hypopharynx, other oral, oesophagus, stomach, colorectal, liver, pancreas,  larynx, lung, melanoma, breast, cervix, uterus, ovary, prostate, testis, bladder, kidney,  brain/central nervous system, thyroid, Hodgkin’s lymphoma, non-Hodgkin’s lymphoma,  multiple myeloma, leukaemia, vulva, vagina, anal, penile and others. | OncoSim-All Cancers model  Using a phase-based costing framework, we  estimated the economic burden of cancer in Canada in 2021 by incorporating published direct health  system costs and patients’ and families’ costs | Out-of-pocket costs, time costs, indirect costs  From a societal perspective, cancer-related costs were CAD 26.2 billion in Canada in 2021; 30% of costs were borne by patients and their families.  The economic burden was the highest in the first year after cancer was diagnosed. During this time, patients and families’ costs amounted to almost CAD 4.8 billion in 2021.  (lung, breast, colorectal, and prostate) had the highest burden | This research findings show that a large proportion of the burden is borne by patients and families (30%), which is often not reported.  This could be informed to cost–benefit analyses of proposed cancer prevention interventions. | 6 |
| 27. | Hong et al. 2019 | USA  The University of Florida, The University of TexasMD Anderson Cancer Centre | In 2011 (n = 655) and in 2016 (n =490)  The sample included adults aged 18–64 with a confirmed diagnosis of cancer  Sample size=1145  Age group:  18-44=224  45-54=344  55-59=252  60-64=335  Sex  Female=778  Male=377  Race/ethnicity  Non-Hispanic White=813  Non-Hispanic Black=130  Hispanic=169  Others=43 | All cancers reported in 2011 & 2016 | Using data from the MEPS-Experiences with Cancer Survivorship Survey (in 2011 and 2016) | There was FT subjective (self-reported financial hardship) and objective (total out-of-pocket expenditure) | Findings suggest the need for the development of  provisions that help cancer patients reduce both perceived  and materialized burden of cancer care. | 6 |
| 28. | Iloabuchi et al. 2021 | USA  West Virginia University | Sample size=26,822  Female=13,946  Male=12,876  (age ≥ 66 years) Age group  66-69=6962  70-74=7833  75-79=5642  ≥80=6385  Ethnic Group:  White=22,438  African American=2373  Others=2011 | Breast cancer=9972 prostate=9373  colorectal cancers=4987 Non-Hodgkin’s Lymphoma=2490  Cancer Stage:  Stage 1=8073  Stage 2=10,803  Stage 3=2903  Stage 4=2587  Unknown=2456 | Data from multiple sources.  retrospective cohort study design with a 12-month baseline & follow-up.  between January 2014 and December 2014, identifying relevant low-value services using ICD9/ICD10 and  CPT/HCPCS codes from the linked health claims and patient out-of-pocket expenditure from Medicare claim files and expressed expenditure in 2016 USD. | Indirect medical cost:  About 29 % of older adults received at least one low-value care procedure during the follow-up period and they had significantly higher mean out-of-pocket expenditures.  One in four older adults with incident cancer received low-value care in 12 months after a cancer  diagnosis. | Excess out-of-pocket expenditure was driven by low-value care, fragmentation of care, and an increasing number of pre-existing chronic conditions. | 5 |
| 29. | Lang et al. 2009 | USA  i3 Innovus, Medford, MA, USA  Bayer Healthcare Pharmaceuticals, Montville, NJ, USA | Sample size=392  (a sample of HCC patients  alive and treated in 1999) | Hepatocellular carcinoma (HCC) | The linked SEERMedicare  Database  Measured the annual economic burden of HCC in the United States, including healthcare costs and lost productivity. | National burden of illness  The annual cost of HCC in the United States is $454.9 million, with per-patient costs of $32,907.  Healthcare costs and lost productivity accounted for 89.2% and 10.8% of the total cost, respectively. | Results exhibit a considerable economic impact of HCC and substantial national spending on this  disease | 4 |
| 30. | Lauzier et al. 2013 | Canada  Hôpital du Saint-Sacrement, Québec, QC | Sample size= 800 women & 391 spouses participated  Age group of women  23-49=228  50-59=319  60-88=253  Age group of spouses:  23-49=122  50-59=149  60-88=120 | Breast cancer  Type of breast cancer  Ductal carcinoma in situ=107  Invasive disease= 693 | Prospective cohort study (longitudinal)  Assessed out-of-pocket costs and wage losses during the first year after diagnosis of early  breast cancer among Canadian women and spouses. | Women’s median net out-of-pocket costs during the year after diagnosis were $1002 (2003 Canadian dollars; mean = $1365; SD = $1238), and 74.4% of these  costs resulted from treatments and follow-up.  Spouses’ median costs were $111 (mean = $234; SD = $320), or 9% of couples’ total expenses. | Overall, out-of-pocket costs from breast cancer for the year after diagnosis are probably not unmanageable for most women. However, some women were at higher risk of experiencing financial burdens resulting from these costs. | 5 |
| 31. | Mao et al. 2017 | China  School of Public Health, Fudan University, Shanghai | Sample size=2091  Gender  Male=1106  Female=985 | Bronchioles & lung, Breast, stomach, colon & rectal cancers | Random sampling, from Urban Employee's Basic Medical Insurance claim database (4 cities in 2008)  Services utilization, medical expenses and out-of-pocket  (OOP) the payment was the metrics collected. | high total expenditure ($1228) but lowest OOP payment ($170) among the 4 cities in China (patients with social insurance) | Insurance reduced the economic burden of insured cancer patients, but it is still necessary to provide further financial protection for  cancer patients in China. |  |
| 32. | Sargazi et al. 2022 | Iran  Few referral hospital and data obtained from GLO-BOCAN 2012 and World Bank | 10 000 patients in 2014  Cervical cancer, Ovarian cancer  Patients, Endometrial Cancer | Cervical cancer  ovarian cancer endometrial cancer (all stages) | To assess the economic burden of gynaecological cancers  Prevalence-based cost of illness methodology | The total cost of GCs in Iran was estimated at $51 million in 2014. The direct costs were $32 million, and indirect costs were $19 million of the total annual cost. The total cost of ovarian cancer was the highest among the 3 cancers. | Knowing that the cost of GCs has a signiﬁcant impact on the burden of disease and imposes an economic burden  on the country could force policymakers to allocate their resource to prevention programs. | 6 |
| 33. | Sasser et al. 2005 | USA, Indiana  DE identified administratively  claims data from seven large employers over a  The 3-year period from 1998 through 2000 | 585,441 individuals  women, age 50–64 years; active employees,  including individuals on short- or long-term disability; | Osteoporosis  Breast cancer:  Cardiovascular disease patients | Descriptive analyses were performed to  allow the comparison of each patient sample to a random sample of female employees, also age 50–64  years | Average annual direct costs were higher (*p* < .001) for female employees treated for OP ($6,259), BrCa ($13,925), or CVD ($12,055) when compared with the random sample ($2,951). In addition, average annual indirect costs associated with OP ($4,039), BrCa ($8,236), and CVD ($4,990) were higher (*p* < .001) than indirect costs for the random sample ($2,292). | OP, BrCa, and CVD occur more frequently in women after menopause, imposing a significant financial burden. | 6 |
| 34. | Seifeldin et al. 1999 | United States  The HCUP includes up to 15 diagnosis  codes from the International Classcjkation  of Diseases, Ninth Revision, Clinical Mod-  ification (ZCD-9-CM) | N=237,754  Population aged 50 years ~60 years. | Colon cancer | To estimate the economic burden of hospitalizations for colon cancer.  To assess the relationship  between risk factors, including age, and  treatment charges and to estimate the  number of hospital admissions for colon cancer through the year 2050. | At -$20,000 per admission, charges averaged >$4.5 billion per year during the 4 years studied. Total annual admission charges increased from $3.90 billion to $5.14 billion over the 4-year period | Interventions to decrease the incidence and mortality of colon cancer are needed and should ultimately reduce associated costs. Until such interventions are developed, the economic burden of colon cancer will continue to increase as the population ages | 5 |
| 35. | Sharp et al. 2018 | Ireland, mixed public-private healthcare system | 493 survivors | Colorectal cancer (Stage 1-4) | To investigate cancer-related financial stress, subjective cancer-related financial strain and their association with health-related quality-of-life in colorectal cancer survivors. | 41% reported cancer-related financial stress and 39%  cancer-related financial strain; 32% reported both financial stress and financial strain | Four in ten colorectal cancer survivors reported an adverse financial impact of cancer.  further research is needed to better understand how  financial distress influences survivors’ quality of life. | 6 |
| 36. | Ting et al. 2020 | Sarawak, Malaysia  Sarawak  General Hospital and Subang Jaya Medical Centre in Malaysia | 429 patients | Urologic cancer patients (prostate cancer, bladder and renal cancer) | To examine the prevalence of financial toxicity (FT) and associated factors among urologic cancer patients  Subjective FT was measured by catastrophic health expenditure (healthcare-cost-to-income ratio greater than 40%) and the Personal Financial Well-being Scale, respectively. | Objective and subjective FT were experienced by 16.1 and 47.3% of the respondents | The significant association between both objective and subjective FT and HRQoL highlights the importance of reducing FT among urologic cancer patients. Subjective FT was found to have a greater negative impact on HRQoL. | 6 |
| 37. | Vallejo-Torres et al. 2014 | England | 8658 new registrations of malignant melanoma and 69 840 new registrations of NMSC in England in 2006 | Skin cancer | To estimate the cost of skin cancer in England, and model future costs up to 2020. | Estimated that costs due to skin cancer were in the range of £106–£112 million in 2008. The cost per case of malignant melanoma was £2607 and £2560, using the bottom-up and top-down approaches, respectively.  The mean cost per case of non-melanoma skin cancer was £889 and £1226, respectively. | Effective prevention of skin cancer might not only reduce the signiﬁcant burden of disease but could also save considerable  resources to the NHS. | 6 |
| 38. | Van Agthoven et al. 2001 | Netherlands | University Hospital Rotterdam and the University Hospital Vrije Universiteit Amsterdam, a selection of 854 study subjects was made from a consecutive number of patients in whom the diagnosis ‘was conﬁrmed  between 1994 and 1996. | Head and neck cancers (cancer in the oral cavity, larynx, or oropharynx) | To calculate the costs of head and neck oncology for reimbursement purposes  To determine unit costs, the micro-costing method is based on a detailed inventory and measurement of resources consumed used. | The costs of diagnosis, treatment, and 2 years of follow-up of patients with a primary tumour were €21 858. For patients with a recurrent tumour, this amount was €27 629. The costs of 10 years of follow-up were €423. In total, the average costs per new patient were €31 829, | This data was primarily meant to be used as a basis for  determining reimbursements for head and neck centres,  but can be applied in any hospital if the number of patients with oral cavity or oropharynx tumours and with laryngeal tumours are known | 6 |
| 39. | Yap et al. 2020 | Malaysia | Sarawak  General Hospital,  461 cancer survivors | Cancer patients (Stage 1-4) | To capture the financial toxicity of Cancer survivors, the Comprehensive Score for Financial Toxicity (COST) instrument | Median COST = 22.0. | Survivors faced greater financial toxicity. | 6 |
| 40. | You et al. 2019 | Seoul, Korea  Two academic medical centres in Seoul | 1,087 patients underwent mastectomy and received follow-up were assessed | Breast cancer patients | medical expenses paid by breast cancer patients for two years after mastectomy  A micro-costing approach from the provider’s perspective, based on a retrospective review of patient medical claim records, | Mean cost USD12,108 in 2003-2008) after 2 years of mastectomy | OOP payment burden on patients was concentrated in the initial phase of treatment, and items not covered by the National Health Insurance caused an additional increase in patients’ burden in the initial phase. | 6 |
| 41. | Azzani et al. 2017 | Malaysia, UMMC | Sample size:138 | Colorectal cancer patients | Direct out-of-pocket  non-medical  indirect cost data  CHE was measured too using the capacity to pay 40% or more | The total 1-year patient cost (both direct and indirect)  increased with the stage of colorectal cancer: RM 6544.5 (USD 2045.1) for stage I, RM 7790.1 (USD 2434.4) for stage II, RM 8799.1 (USD 2749.7) for stage III and RM 8638.2 (USD 2699.4) for stage IV. CHE=47.8% | Despite the high subsidisation in public hospitals, the management of colorectal cancer imposes a substantial  the financial burden on patients and their families. | 5 |
| 42. | Kasahun et al. 2020 | Ethiopia | Sample size: 352  cost estimation:2018 | Any type of cancer | Medical and non-medical expenses form the patient’s perspective | Mean medical cost: $1978 (median: $1394), mean non-medical cost: $388 (median: $222) | A substantial number of patients with cancer were exposed to CHE with considerable medical expenditure. financial risk protection and realising universal health coverage for patients with cancer is crucial. | 5 |
| 43 | Albelda et al. 2019 | USA | Sample size: 171 | Bone marrow transplant among cancer patients | Cross-sectional study for 6 months after diagnosis  Subjective FT using 3 questions:  how satisfied are you with your family’s present financial situation?” (1 = completely satisfied; 5 = not satisfied at all); “How difficult is it for you/your family to meet monthly payments on your bills” (1= not difficult at all, 5= extremely difficult); and “How do your family’s finances usually work out at the end of the month?” (1=some money left over, 2=just enough money, 3=not enough money). | 9% answered not at all for Q1, 6 % answered extremely difficult for Q2 and 18% answered not enough for Q3 | These findings suggest universal paid leave policies in the United States might alleviate financial hardship | 5 |
| 44 | Banegas et al. 2016 | USA | Sample size:4719 | Any type of cancer | Cross-sectional,  Assess the subjective FT using 4 questions: 1. worrying about having to pay large bills related to their cancer, 2. they or someone in the family had gone into debt because of cancer, 3. they or their families had filed for bankruptcy 4. making other financial sacrifices | sixty-four percent reported worrying about having to pay large bills related to their cancer. Thirty-four percent reported that they or someone in the family had gone into debt because of cancer, and 3 percent said that they or their families had filed for bankruptcy as a result of cancer. Forty percent reported making other financial sacrifices. | Stakeholders have to develop evidence-based interventions and policies to reduce the financial hardship of cancer. | 5 |
| 45 | Casilla-Lennon et al. 2018 | USA | Sample size: 138 | Bladder cancer | Selecting “agree” or “strongly agree” on the following statement; “You have to pay more for medical care than you can afford” | 24% reported FT | Financial toxicity is a major concern among patients with bladder cancer. | 4 |
| 46 | Finkelstein et al. 2009 | USA | Sample size: 1940  Medical Expenditure Panel Survey | Cancer of any type | National OPP and loss of productivity | OOP during active cancer stage is $1730 and 1180 in follow up stage. 22.3 days are the average length of loss of productivity | The results reveal the financial implications that are likely to result from a cancer diagnosis are substantial | 5 |
| 47 | Gordon et al. 2017 (a) | Australia | Sample size: 289 | Prostate cancer | OOP of patients | OOP median is AU$8000 | The findings suggest a large variability in medical costs for prostate cancer treatment and due to out‐of‐pocket expenses, some men facing very high costs. | 5 |
| 48 | Gordon et al 2017 (b) | Australia | Sample size: 187  Data collected in Jan 2010 to Sep. 2011 | Colorectal cancer | FT at 6- and 12-months following diagnosis using FT questionnaire of 3 domains: perceived prosperity, financial strain and ability to raise money | 1% to 0.6% answered as poor for 1^st^ domain at 6 and 12 months, financial strain reported by 15% and 7% at 6 and 12 months, difficult to raise money 41% and 33% at 6 and 12 months, respectively | Financial Toxicity should be addresses among colorectal cancer patients | 6 |
| 49 | Gordon et al. 2007 | Australia | Sample size: 287 | Breast cancer | population-based study of 287 women was used to explore economic outcomes (costs and lost income) for women with breast cancer 0-18 months post-diagnosis. Survey methods collected data on out-of-pocket costs, care-giving support, paid and unpaid work reductions, and perceptions from participants on these financial impacts. | Women direct mean cost is US$1937 and mean indirect cost is US$6093 | Economic research adds an important dimension for understanding the impact of breast cancer, and findings may be used to help improve supportive care services for women and families confronted by this disease. | 6 |
| 50 | Guerin et al. 2016 | USA | Sample size: 132 | Brain metastasis among lung cancer | Direct and indirect cost was assessed from payer perspective | Mean direct cost $86,027 and mean indirect cost is $8528 | the cost of patients’ productivity loss following the development of brain metastasis increased dramatically | 5 |
| 51 | Massa et al. 2019 | USA | Sample size 16,771 | Head and neck cancers in compare to other types of cancer | Direct cost was assessed | Median annual medical expenses ($8384 vs $5978; difference, $2406; 95% CI, $795-$4017) | The financial strain on individuals, assessed as relative out-of-pocket expenses, appears to be driven more by income than by health factors, and health insurance does not appear to be protective. | 4 |
| 52 | Guy et al. 2013 | USA, Centers for Disease Control and  Prevention; Emory University, Atlanta,  National Cancer Institute,  Bethesda, Dowling, Institute for Technology  Assessment, Massachusetts General  Hospital, Boston, MA. | Sample size: 4,960 | All types (Mixed) | MEPS, direct medical costs were estimated using total annual medical expenditures, by source of payment and service type | Older age and higher household savings were negatively associated with financial toxicity; non-regular employment, retirement because of cancer, and use of strategies to cope with the cost of cancer care were negatively associated with COST score. | Cancer survivors aged 18 to 64 years were more likely to report employment disability, and higher number of missed workdays because of health, and higher number of additional days spent in bed because of health than individuals without a history of cancer. Efforts to reduce the economic burden caused by cancer will be increasingly important  given the growing population of cancer survivors. | 5 |
| 53 | Honda et al. 2019 | Japan, Aichi Cancer Center Hospital, Nagoya, Japan | Sample size: 156 | All types (Solid tumours) | Comprehensive Score for Financial Toxicity (COST) tool to measure financial toxicity (FT), OOP medical costs, total family income, and total family savings (collected by using a questionnaire and medical  Records) | Median COST score was 21 (range, 0 to 41; mean ± standard deviation, 12.  1 ± 8.45), with lower COST scores indicating more severe FT. On multivariable analyses using linear regression, older age (β, 0.15 per year; 95% CI, 0.02 to 0.28; P = .02) and higher household savings (β, 8.24 per ¥15 million;  95% CI, 4.06 to 12.42; P,.001) were positively associated with COST score; nonregular employment (β, −5.37; 95% CI, −10.16 to −0.57; P = .03), retirement because of cancer (β, −5.42; 95% CI, −8.62 to −1.37; P = .009), and use of strategies to cope with the cost of cancer care (β, −5.09; 95% CI, −7.87 to −2.30; P , .001) were negatively associated with COST score. | Various factors associated with FT in  Japanese patients with cancer was identified. These findings will have important implications for cancer policy planning in  Japan. | 5 |
| 54 | Huntington et al. 2015 | USA, Division of Hematology-Oncology, Division of Internal  Medicine and the Abramson Cancer Center and Division of General Internal Medicine,  University of Pennsylvania,  Philadelphia, PA, USA | Sample size: 100 | Multiple myeloma | COST measure | 59 (59%) of 100 patients reported that treatment costs were higher than expected, 70 (71%) of 99 had at least minor financial burden, and 36 (36%) of 100 reported applying for financial assistance. Use of savings to (43 [46%] of 94 patients) and 21 (21%) of 98 individuals borrowed money. COST scores were highly correlated with patient-reported use of strategies to cope. On multivariable analysis, younger age, non-married status, longer duration since diagnosis and lower household income were associated with higher financial burden. | financial toxicity and use of coping mechanisms were common in the insured  population with multiple myeloma. Additional attention to rising treatment costs and cost sharing is needed to address the increasing evidence of financial toxicity affecting patients with cancer. | 6 |
| 55 | Jagsi et al. 2014 | USA, University of  Michigan, Virginia Commonwealth University, University of Southern California, and Cancer Institute of  New Jersey, Robert Wood Johnson Medical  School. | Sample size: 1,502 | Breast cancer | Longitudinal study (2005 to 2007) from SEER registries of metropolitan Los Angeles and Detroit.  Multivariable models evaluated correlates of self-reported decline in financial status attributed to breast cancer and of experiencing  at least one type of privation  Questionnaires were based on existing literature, measures previously developed to assess relevant constructs, and theoretical models. | median out-of-pocket expenses were  ≤ $2,000; 17% of respondents reported spending > $5,000; 12% reported having medical debt 4 years postdiagnosis. Debt varied significantly by race: 9% of whites, 15% of blacks, 17% of English-speaking Latinas, and 10% of Spanish-speaking Latinas reported debt (P = .03). Overall, 25% of women experienced financial decline at least partly attributed to breast cancer;  At least one privation was experienced by 18% of the sample. | Racial and ethnic minority patients appear most vulnerable to privations and financial decline  attributable to breast cancer, even after adjustment for income, education, and employment.  These findings should motivate efforts to control costs and ensure communication between patients and providers regarding financial distress, particularly for vulnerable subgroups. | 5 |
| 56 | Inguva et al. 2022 | USA, University of Mississippi School of Pharmacy | Sample size: | All types (Mixed) | 2016-2017 Medical Expenditure Panel Survey data used to find cancer-related financial toxicity. Multivariable regression analyses employed to examine the association between cancer-related financial toxicity and cancer survivors' self-reported physical and mental health outcomes and caregiver burden. | 53.7% of adult cancer survivors reported experiencing financial toxicity. Those who experienced financial toxicity reported 14% greater pain, and poorer physical and mental health outcomes as compared to those who did not experience financial toxicity | Intervention programs for reducing financial toxicity among adult cancer survivors and their caregivers should be developed. | 5 |
| 57 | Kale et al. 2016 | USA, Division of Pharmacoeconomics and Health Outcomes, School of Pharmacy, Virginia Commonwealth University, | Sample size: 19.6 million | All types (Mixed) | Cross-sectional study Analyzed data using the 2011 Medical Expenditure  Panel Survey (MEPS), which included the Cancer Self-  Administered Questionnaire (CSAQ)  The following outcomes were evaluated: Physical Component Score (PCS) and Mental Component Score (MCS) of the 12-Item Short-Form Health Survey (SF-12), depressed mood, psychological distress, and worry related to cancer recurrence. The authors also assessed the effect of the number of financial problems  on these outcomes. | 28.7% reported financial burden. Among them, the average PCS (42.3  vs 44.9) and MCS (48.1 vs 52.1) were lower for those with financial burden versus those without. In adjusted analyses, CS with financial  burden had significantly lower PCS (b5-2.45), and MCS (b5-3.05), had increased odds of depressed mood (odds ratio, 1.95), and  were more likely to worry about cancer recurrence (odds ratio, 3.54). Survivors reporting ≥3 financial problems reported statistically significant and clinically meaningful differences (≥3 points) in the mean PCS and MCS compared with survivors without financial problems. | Cancer-related financial burden was associated with lower health-related quality of life, increased risk of  depressed mood, and a higher frequency of worrying about cancer recurrence among CS. | 4 |
| 58 | Rogers et al. 2012 | UK, Faculty of Health, Edge Hill University, University Hospital Aintree, School of Medicine, University of Liverpool | Sample size: 447 | Head and Neck cancers | University Hospital Aintree head and neck cancer database (Jan-Dec 2008). The Cost of Head and Neck Cancer Questionnaire (postal) | The most notable financial costs that were a moderate or large burden to patients were petrol (25%, 112), home heating (24%, 108), change in the type of food (21%, 95), and loss of earnings (20%, 88).  15% (63/423) had lost a moderate or large amount of income because of their medical condition. In terms of taking care of their financial needs, 10% (40) were moderately dissatisfied and 15% (61) very dissatisfied. Patients with worse physical and social emotional functioning  experienced more notable financial burden | Cancer of the head and neck has a serious impact on financial aspects of patients’ lives and associated with a poor HRQoL. Multidisciplinary teams can do much more to address the cost of having treatment by recognising need earlier and giving advice and access to appropriate benefits. | 5 |
| 59 | Shankaran et al. 2012 | USA, National Cancer Institute, 2008-2010 | Sample size:  284 out of 555 were eligible with a response rate of 51.2%.  Mean age 59.5 years | Stage III colon cancer | Objective: to determine financial hardship factors in resected stage III colon cancer patients getting adjuvant treatment.  Multidimensional survey instrument was used | 38% of cancer patients had financial hardships, 23% were in debt with an average debt of $26,860, and 27% had to sell stocks or use savings or retirement funds.  Younger age, nonwhite race, lower annual income, Medicaid or lack of insurance, and work disability, leave-of-absence, or unemployment were significantly associated with financial hardship in univariate analysis. | Many stage III colon cancer patients receiving adjuvant treatment may struggle financially. Helping these people throughout therapy could prevent long-term financial issues. | 6 |
| 60 | Sharp and Timmons, 2016 | Ireland, National Cancer Registry. | 698 of 740 survivors (54%) were analysed.  Exclusion: 19 did not answer whether cancer caused FT, 20 had lung cancer, and three men had breast cancer;  Mean age was 57.5 years  Postal questionnaires were distributed to 1373 people  diagnosed with cancer 3–24 months previously identified  from the National Cancer Registry Ireland | Breast, prostate, and lung cancer  212 (30 %) were men with prostate  cancer and the remaining 486 (70 %) women with  breast cancer | Objective : to identify predictors of cancer-related financial hardship in survivors of female breast cancer and prostate cancer.  A questionnaire assessing the financial and economic consequences of cancer was used | Nineteen percent had financial stress before diagnosis.  Three-quarters had direct medical out-of-pocket costs (mean=€1491, standard deviation=€4053); 87% had other cancer-related costs (mean=€1180, standard deviation=€7559); and 57% indicated that their household spending had increased due to cancer.  48% reported cancer-related financial hardship and 32% strain.  Significant predictors of cancer-related financial stress were: prediagnosis employment position; whether the survivor had dependants; sex; medical card and private health insurance status; household income and financial stress; direct medical expenditures and whether the survivor has higher family bills owing to cancer. | Financial stress affects many Irish breast and prostate cancer survivors. Prediagnosis employment and finances indicate post-diagnosis financial well-being.  These findings may help build methods to identify patients/survivors who need financial guidance and support. | 6 |
| 61 | Wheeler et al. 2018 | USA  Carolina Breast Cancer Study  from 2008 to 2013 | 2,494 women (49% black, 51% white) were included from 2,998 study participants. We excluded women who reported a race other than black or white (n = 82), women who did not complete the follow-up survey (n = 422) due to death (120) or nonresponse (302). | Breast cancer, all stages | Objective: To examine breast cancer's financial burden by race in a large population-based prospective cohort study.  Rapid case ascertainment through the North Carolina state cancer registry enrolled women from 2008 to 2013 across 44 counties at diagnosis. a National Cancer Institute-modified model that describes direct and indirect causes of financial impact. | 58% of black women experienced financial hardship since diagnosis, compared to 39% of white women (P,.001). In models adjusted for age, stage at diagnosis, and therapy received, black women were more likely to report adverse financial effect attributed to cancer, including income loss, healthcare–related care–related financial barriers, health care–related transportation barriers, and job loss. Race's effect was reduced by socioeconomic characteristics but remained substantial for job loss, transportation hurdles, income loss, and overall financial impact. | Compared with white women, black women with breast cancer experience a significantly worse  financial impact. Disproportionate financial strain may contribute to higher stress, lower treatment  compliance, and worse outcomes by race. Policies that help to limit the effect of cancer-related  financial strain are needed. | 6 |
| 62 | Whitney et al. 2015 | USA | 1209 cancer survivors .  Data were derived from the 2011 Medical Expenditure Panel  Survey Household Component (MEPS-HC) and Experiences  with Cancer Survivorship Supplement (ECSS). | Various cancer types and stages , except melanoma of the skin | to examine predictors of cancer-related financial difficulties and work modifications in a national sample of cancer survivors  National survey was used | 33.2% of survivors indicated financial concerns, with 17.9% reporting debt or bankruptcy. 44.0% of working survivors made work adjustments, 15.3% of which were long-term (e.g., delayed or early retirement). Race/ethnicity other than white, non-Hispanic, income <200% of federal poverty level (FPL) or between 200 and 400% of FPL, residence in a non-metropolitan service area , and good/fair/poor self-rated health were predictors of financial difficulty include. Good/fair/poor self-rated health, being married, uninsured, or publicly insured were predictors of long-term work changes in survivors under 65 years old. | The study provides information on US cancer survivors' financial and professional changes. Updated national figures show the need to address the work and financial burden of cancer treatment on survivors from active treatment through long-term survivorship. | 6 |
| 63 | Yabroff et al. 2016 | USA, | Sample size: 1,202 18-year-old adult cancer survivors from the 2011 Medical Expenditure Panel Survey Experiences With Cancer questionnaire. | Not specified | Objective: To determine cancer-related financial hardship in the US and identify cancer survivors experiencing financial hardship.  Material and Psychological financial hardship questionnaires were used. | Material and psychological financial difficulties were more common in cancer survivors aged 18–64 than those 65 and older. In adjusted analyses, cancer survivors aged 18–64 who were younger, female, nonwhite, treated more recently, and had changed jobs due to cancer were considerably more likely to experience financial difficulties. Uninsured, lower-income, and recently treated cancer survivors reported increased psychological financial difficulty. Younger cancer survivors under 65 reported increased financial difficulties. | Financial difficulty is frequent for cancer survivors, especially working-age ones. | 6 |
| 64 | Zheng et al. 2015 | Portland, USA | The 2008 to 2012 Medical Expenditure Panel Survey data were used , which is a nationally representative  survey of the US civilian noninstitutionalized population  conducted by the Agency for Healthcare Research and Quality  Sample size: colorectal (n = 540), female breast (n = 1568), and prostate (n = 1170) cancer survivors and individuals without a cancer history (n = 109 423). Response rates ranged from 53.5% to 59.3% from 2008 to 2012. The final sample consisted of colorectal (nonelderly: n = 169; elderly: n = 371), breast (nonelderly: n = 777; elderly: n = 791), and prostate (nonelderly: n = 281; elderly: n = 889) cancer survivors and individuals without a cancer history (nonelderly: n = 95 640; elderly: n = 13 792). | Colorectal, Breast and prostate cancers | Objective :  To estimate the annual economic burden among survivors of the three most prevalent cancers (colorectal, female breast, and prostate) in both nonelderly and elderly populations in the United States. | Compared with individuals without a cancer history, cancer survivors experienced annual excess medical expenditures (for the non-elderly population, colorectal: $8647, 95% confidence interval [CI] = $4932 to $13 974, P < .001; breast: $5119, 95% CI = $3439 to $7158, P < .001; prostate: $3586, 95% CI = $1792 to $6076, P < .001; for the elderly population, colorectal: $4913, 95% CI = $2768 to $7470, P < .001; breast: $2288, 95% CI = $814 to $3995, P = .002; prostate: $3524, 95% CI = $1539 to $5909, P < .001).  Cancer survivors in the US experienced higher medical expenditures and productivity losses compared to those without cancer. Colorectal cancer survivors had the highest annual medical expenditures and productivity losses, followed by female breast cancer and prostate cancer survivors. | Colorectal, breast, and prostate cancer survivors had statistically significantly higher economic burden than people without a cancer history, but the burden varied by disease site and age. Colorectal, breast, and prostate cancer costs can be reduced by targeted programmes. | 6 |

**Search database**

1. **Database:** Pubmed

Date of search:15.8.2022

Total results:50

Keywords:

Search: **((((treatment) AND "healthcare") AND "cancer") AND "cost of illness") AND ((("Financial hardship") OR "financial toxicity") OR "financial burden")**

("therapeutics"[MeSH Terms] OR "therapeutics"[All Fields] OR "treatments"[All Fields] OR "therapy"[MeSH Subheading] OR "therapy"[All Fields] OR "treatment"[All Fields] OR "treatment s"[All Fields]) AND "healthcare"[All Fields] AND "cancer"[All Fields] AND "cost of illness"[All Fields] AND ("Financial hardship"[All Fields] OR "financial toxicity"[All Fields] OR "financial burden"[All Fields])

1. **Database:** ScienceDirect

Date of search:28.8.2022

Total results: 59

((((treatment) AND "healthcare") AND "cancer") AND "cost of illness") AND ((("Financial hardship") OR "financial toxicity") OR "financial burden")

1. **Database:** Scopus

Date of search: 28.8.2022

Total results: 40 Date of search: 28.8.2022

((((treatment) AND "healthcare") AND "cancer") AND "cost of illness") AND ((("Financial hardship") OR "financial toxicity") OR "financial burden")

1. **Database:** CINAHL

Date of search: 28.8.2022

Total results: 95

((((treatment) AND "healthcare") AND "cancer") AND "cost of illness") AND ((("Financial hardship") OR "financial toxicity") OR "financial burden")
